# Autophagy is dysregulated in spinocerebellar ataxia type 2

**DOI:** 10.1080/27694127.2022.2065603

**Published:** 2022-04-20

**Authors:** Adriana Marcelo, Carlos A. Matos, Clévio Nóbrega

**Affiliations:** a ABC-RI, Algarve Biomedical Center Research Institute, Algarve Biomedical Center, Faro, Portugal; b Faculty of Medicine and Biomedical Sciences, University of Algarve, Faro, Portugal

**Keywords:** Autophagic activation, autophagy, cordycepin, neurodegeneration, polyglutamine diseases, spinocerebellar ataxia type 2.

## Abstract

The accumulation of misfolded proteins and fibrillar aggregates inside neurons is a hallmark of several neurodegenerative diseases. While the exact role of these aggregates is still controversial, they are part of a cascade of molecular events that underlies the neuronal dysfunction and degeneration observed in these disorders. In this line, multiple studies on pathological events involved in neurodegenerative diseases have reported dysfunction of quality control mechanisms. In fact, the inhibition of macroautophagy/autophagy in the CNS per se, in the absence of additional stress stimuli, is sufficient to induce neurodegeneration. For neuronal homeostasis to be maintained, protein clearance systems must function properly, allowing the degradation of damaged organelles and pathogenic misfolded proteins. Therefore, in the last years there has been a strong focus on the study the dysregulation of protein clearance systems, especially autophagy, in the context of neurodegenerative diseases.

## Autophagy and polyglutamine diseases

Polyglutamine (polyQ) diseases are a group of nine inherited neurodegenerative conditions caused by abnormal expansions of CAG/glutamine repeats in the genes/proteins associated with each disorder. Although these genetic causes are well-established, the precise events that trigger the pathogenic alterations associated with polyQ diseases are still debatable. In line with other neurodegenerative diseases, multiple studies have reported a dysregulation in the autophagic process in these disorders. However, for spinocerebellar ataxia type 2 (SCA2), few studies have addressed the status of autophagy and its implication in the disease. Therefore, in our recent study, we aimed to clarify the role of autophagy in SCA2 pathogenesis.

## Autophagy dysregulation in SCA2

To examine the autophagy pathway in SCA2 we used different disease models: (i) mouse neuroblastoma (Neuro2a) cells expressing the causative mutant protein (expanded ATXN2 [ataxin 2]); (ii) a new SCA2 lentiviral mouse model with striatal pathology, generated and characterized in this study; and (iii) striatal and cerebellar patient-derived post-mortem tissues [1]. We analyzed the expression of the autophagy markers SQSTM1, which binds to ubiquitinated substrates and targets them to phagophores, and LC3, a protein that plays a role in autophagosome maturation.

In Neuro2a cells expressing expanded ATXN2, we found that SQSTM1 protein levels are increased, and LC3B-II levels are decreased, compared with cells expressing non-expanded ATXN2. Accordingly, in the new SCA2 lentiviral mouse model we find higher mRNA and protein levels of *Sqstm1*/SQSTM1 upon expression of expanded ATXN2. Alterations in the levels of these two proteins suggest malfunctioning key steps of the autophagic pathway. SQSTM1 is cleared by autophagy, but not by the ubiquitin-proteasome system (UPS), and thus the accumulation of this marker can imply an ineffective lysosomal degradation and autophagy inhibition. Additionally, increased levels of SQSTM1 can compromise UPS function, by delaying the delivery of ubiquitinated substrates to this complex, exacerbating the buildup of toxic components inside neurons. Because LC3 is required for autophagosome formation and maturation, an increase or a decrease in its levels may suggest a dysfunction in autophagy.

We further investigated the expression pattern of these two autophagy markers in SCA2 patients’ post-mortem samples of cerebellum and striatum, two brain regions reported to be affected in the disease. Histological analysis reveals an abnormal accumulation of SQSTM1 and LC3 in aggregate-like inclusions in both tissues, representing important evidence of autophagic impairment in the brain of SCA2 patients. The accumulation of these autophagic vesicles has been observed in other neurodegenerative diseases, suggesting decreased autophagic flux. Overall, our results suggest that the autophagic pathway is dysfunctional in SCA2, which is in line with what has been reported for the other polyQ diseases.

## A new target for therapeutic intervention in SCA2

In the last years, autophagy became one of the most promising and studied targets for therapeutic intervention in neurodegenerative diseases, including polyQ diseases. In this context, we assessed whether pharmacological activation of autophagy can rescue SCA2-associated neuropathological abnormalities observed in the newly developed lentiviral mouse model. For that, we used the compound cordycepin, which we have shown previously to promote autophagy and mitigate the phenotypic alterations in models of the polyQ disorder Machado-Joseph disease/MJD. In accordance with that study, we observed that treatment with cordycepin results in fewer aggregates of expanded ATXN2 in both cellular and mouse models of SCA2. Moreover, cordycepin administration leads to a reduction of neuropathological signs *in vivo*, including of neuronal loss, neuronal death and neuroinflammation. Future studies using this compound or other autophagy activators should be conducted in other SCA2 animal models.

## Future perspectives

**Figure 1.**
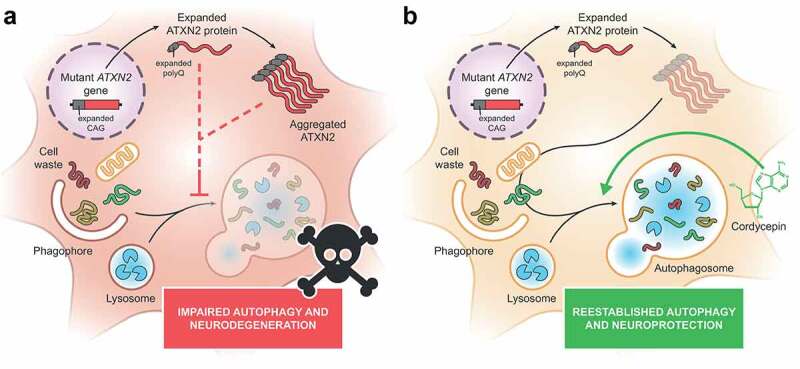
Dysregulation of autophagy is a feature of spinocerebellar ataxia type 2 (SCA2) neuropathology. (**A**) In SCA2, the mutant *ATXN2* is expressed as an abnormally expanded ATXN2 protein, which tends to aggregate in the cytoplasm of neurons from select brain regions. Evidence collected from cell cultures, an animal disease model and patient brain samples suggests that autophagy is compromised in the context of SCA2, as a result of expanded ATXN2 expression. (**B**) Stimulating autophagy through cordycepin administration produces amelioration of diverse neuropathological features of an *in vivo* SCA2 model, reducing protein aggregation and neuronal death.

Overall, our study presents clear evidence for an impairment of autophagy in SCA2 and demonstrates that pharmacological upregulation of this pathway produces a neuroprotective effect in SCA2 disease models (Figure 1). The potential therapeutic effect of autophagic modulators is reinforced by several studies evaluating this strategy in other polyQ diseases. These studies have used either pharmacological approaches or gene therapies to enhance autophagy in different disease systems, reporting very promising results. Nonetheless, some issues regarding autophagy activation should be tackled, such as the effectiveness of some autophagy modulators that failed to attain the expected beneficial effects in pre-clinical and clinical studies, the ability to specifically target damaged tissues, without affecting regions that are spared in the diseases, and the ability to elicit autophagy activation in a controlled way, producing no toxic effects.

## References

[1] Marcelo, A., Afonso, I.T., Afonso-Reis, R. *et al*. Autophagy in Spinocerebellar ataxia type 2, a dysregulated pathway, and a target for therapy. *Cell Death Dis* 12, 1117 (2021). 34845184 10.1038/s41419-021-04404-1 PMC8630050

